# ﻿Aedeagal sensilla of *Agelastica alni* (Coleoptera, Chrysomelidae, Galerucinae)

**DOI:** 10.3897/zookeys.1252.148748

**Published:** 2025-09-19

**Authors:** Michael Schmitt

**Affiliations:** 1 Universitaet Greifswald, General and Systematic Zoology, Loitzer Str. 26, D-17489 Greifswald, Germany Universitaet Greifswald Greifswald Germany

**Keywords:** Aedeagus, alder leaf beetle, chemosensor, male copulatory organ, mechanosensor, morphology

## Abstract

The intromittent part – the median lobe – of the male copulatory organ – the aedeagus – of beetles is strongly sclerotised and so could give the impression of being just a rigid tube, not providing any sensory input to the male beetle. However, it has already been shown that in many beetle species, the median lobe bears sensilla of different types and in different densities. Here, the morphology of the cuticular elements of putative sensilla on the median lobe of the alder leaf beetle, *Agelastica alni* (Linnaeus, 1758) and their spatial distribution on this organ are described. There are especially two types of sensilla located in large numbers on the median lobe. One is mamilliform and is probably a bimodal uniporous sensillum, the other is cup-shaped with a pore on top suggesting a contact chemosensor. In addition, there are numerous – presumably mechanosensitive - sensilla basiconica. The density of these sensilla is higher apically and on the lower side than basally and on the upper side of the median lobe. These findings confirm earlier ideas that the male copulatory organs in beetles are not sensory-blind but are sensitive to mechanical and chemical stimuli.

## ﻿Introduction

The morphology of the male copulatory organs in beetles is conspicuously more complicated and diverse than the transfer of sperm from male to female would technically require, as for example, discussed in length by Eberhard (1985). Thus, they also serve other functional roles than just transferring sperm. Observations of mating behaviour of beetles (e.g., Düngelhoef and Schmitt 2006; Eberhard 2023) indicate that male beetles can use their intromittent organs to perceive at least tactile stimuli from the female genital tract. Up to now, only scattered information on the sensory equipment of the male copulatory organs of beetles is available. Zacharuk (1985: 22) wrote “Sensilla on the male genitalia have received little attention”, and still in 2012, Faucheux found that for Coleoptera “literature concerning the male genitalia is less common” with respect to sensilla and could only list nine titles (Hammond 1972; Epila-Otara and Triplehorn 1990; Kima and Yamasaki (1996); Kim et al. 1999; Kim and Sota 2004, 2006; Hubweber and Schmitt 2006; Düngelhoef and Schmitt 2006, 2010). In these studies, different types of sensilla on the median lobe of the aedeagus are described, mostly peg-like and campaniform sensilla. Consequently, one could expect that male beetles are indeed able to perceive mechanical stimuli during copulation through the physical contact of their intromittent organs with the female genital tract. These sense organs are probably involved in sperm transfer in the sense that they presumably aid the male in achieving intromission, for example, by sensing that the female has opened her external genital opening, a requirement for intromission to occur in other chrysomelid species that have been studied (Eberhard and Kariko 1996). In addition, they could play a part in internal courtship or stimulation of the female.

The aim of the present study is to check what types of sensilla are found on the median lobe of the aedeagus in the alder leaf beetle *Agelastica alni* (Linnaeus, 1758), count the number of these sensilla and map their distribution on the median lobe, and thus get an idea of what a male beetle can perceive during copulation. The alder leaf beetle was chosen as the study species because it is easily accessible and males have a simple aedeagus as compared to beetles with an aedeagus with tegminal parameres, like Curculionoidea, Cerambycidae, and some Chrysomelidae: Donaciinae, Sagrinae, Bruchinae, and Timarchini.

## ﻿Material and methods

Alder leaf beetles were collected in the vicinity of Greifswald (North-East Germany). Nine males were dissected, their aedeagi cleaned mechanically by ultrasound and chemically in potassium hydroxide solution and mounted on scanning electron microscope (SEM) stubs. The probes were sputter coated with gold-palladium (Polaron Range SC7620, Quorum Technologies) and analysed under a SEM, seven of them in a normal SEM Evo LS10 (Carl Zeiss), two using a high-resolution SEM (field emission, FE-SEM), model Leica EM CPD300.

**Figure 1.**
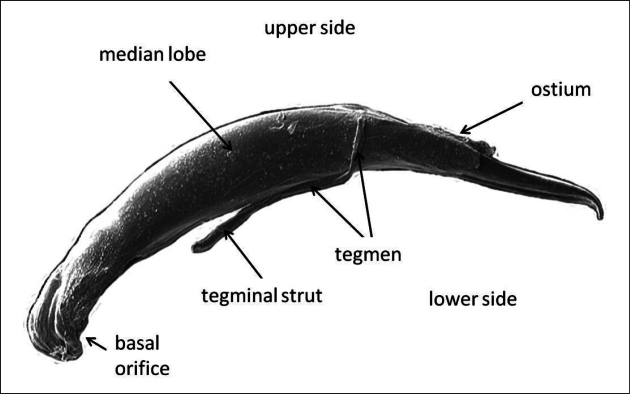
SEM photo of an aedeagus of *Agelastica alni* showing the terms of orientation as used in the text.

The aedeagus is positioned inside the male abdomen lying on one side and is turned by 90° when protruded. During copulation, the male bends his abdomen and inserts the median lobe of the aedeagus into the female genital opening with the upper side facing down. Thus, application of the terms “dorsal” and “ventral” could lead to misunderstandings. Therefore, the terms “upper side” and “lower side” are used here as shown in Fig. 1.

Hand drawings of the aedeagi on the basis of SEM photos were edited using the graphics programmes GIMP v. 2.8.14, Adobe Photoshop Lightroom v. 5.3 and Microsoft Paint.

## ﻿Results

**Figure 2.**
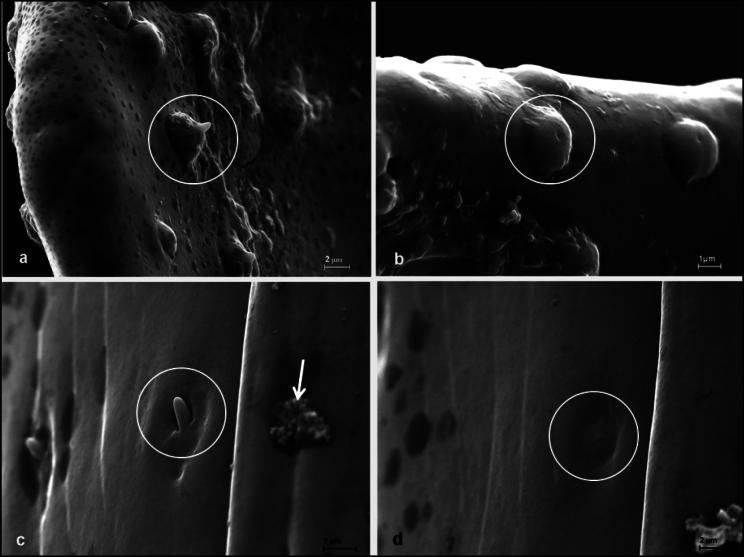
(Putative) sensilla on the median lobe (circled). **a.** Sensillum mamilliforme (centre) on the lower right border of the median lobe near the apex; **b.** Sensilla cupuliforma on the rim near the apex; **c.** Putative sensillum basiconicum (centre) on the lower side of the apex region. A glandular opening in the right half of the photo (arrow) is covered with some excretion; **d.** Possibly a sensillum campaniforme, near c. The dark disk-shaped areas are probably caused by lipid droplets under the surface coating (see Schertel et al. 2013: 357).

Numerous sensilla were found on the median lobe, which could not be easily assigned to the sensilla types described by Altner (1977). Especially, two kinds of putative sensilla do not fit into the traditional classifications. One is characterised by a roundish mount with a conical extension at the highest point (Fig. 2a) and seemingly sitting in a ring-shaped depression. The diameter of the base is c. 4 µm, its maximal height 3 µm. The height is greatly variable – many of these sensilla are only slightly elevated (e.g., Fig. 6a), some appear even depressed (Fig. 6b). Although the sensilla of this type showed considerable variation in shape, I use “mamilliform” to describe them. This means that the term “sensillum mamilliforme” (plural: s. mamilliformia) refers to the outer appearance only and is used here just as a label. The putative sensilla of the other type, not corresponding to the textbook-categories, look like bumps with a central pore (Fig. 2b). They are c. 3 µm wide and between 0.5 and 1.5 µm high. These sensilla are tentatively labelled “sensilla cupuliformia” here. Besides these putative sensilla, there are also structures resembling sensilla basiconica (Fig. 2c). Sensilla of these three types were found on all studied specimens. Only on one median lobe was a structure that could be a sensillum campaniforme (Fig. 2d).

**Figure 3.**
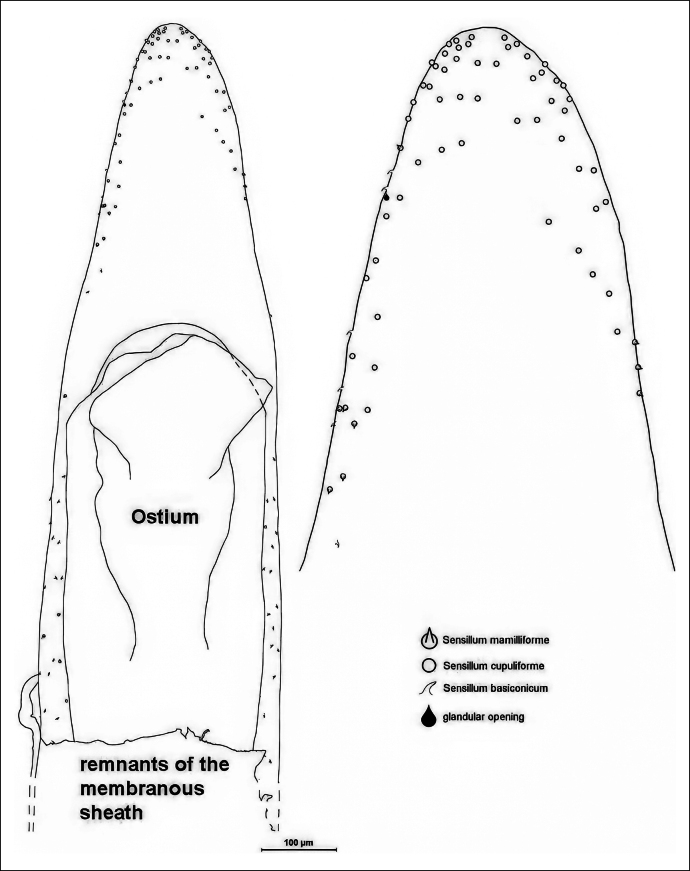
Distribution of sensilla on the upper side of the median lobe of one specimen. On the right, the apical region is enlarged to make the symbols recognisable. The symbols are to mark the sites; the sizes of the symbols are not proportional to the true sizes of the structures.

**Figure 4.**
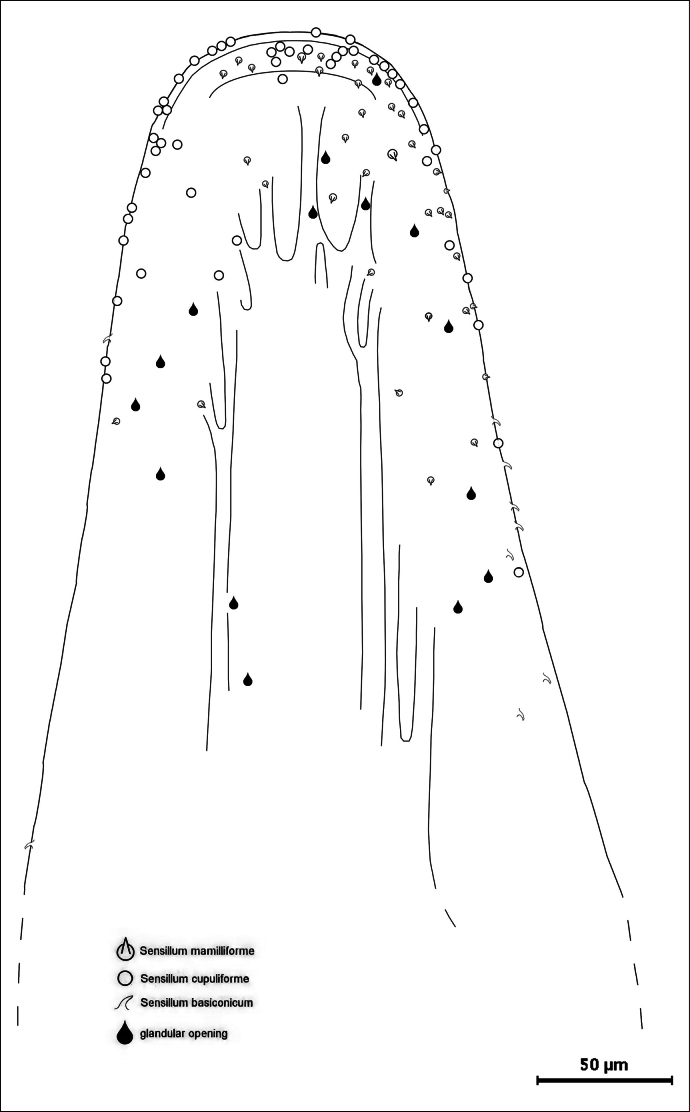
Distribution of sensilla on the lower side of the apical region of a median lobe.

**Figure 5.**
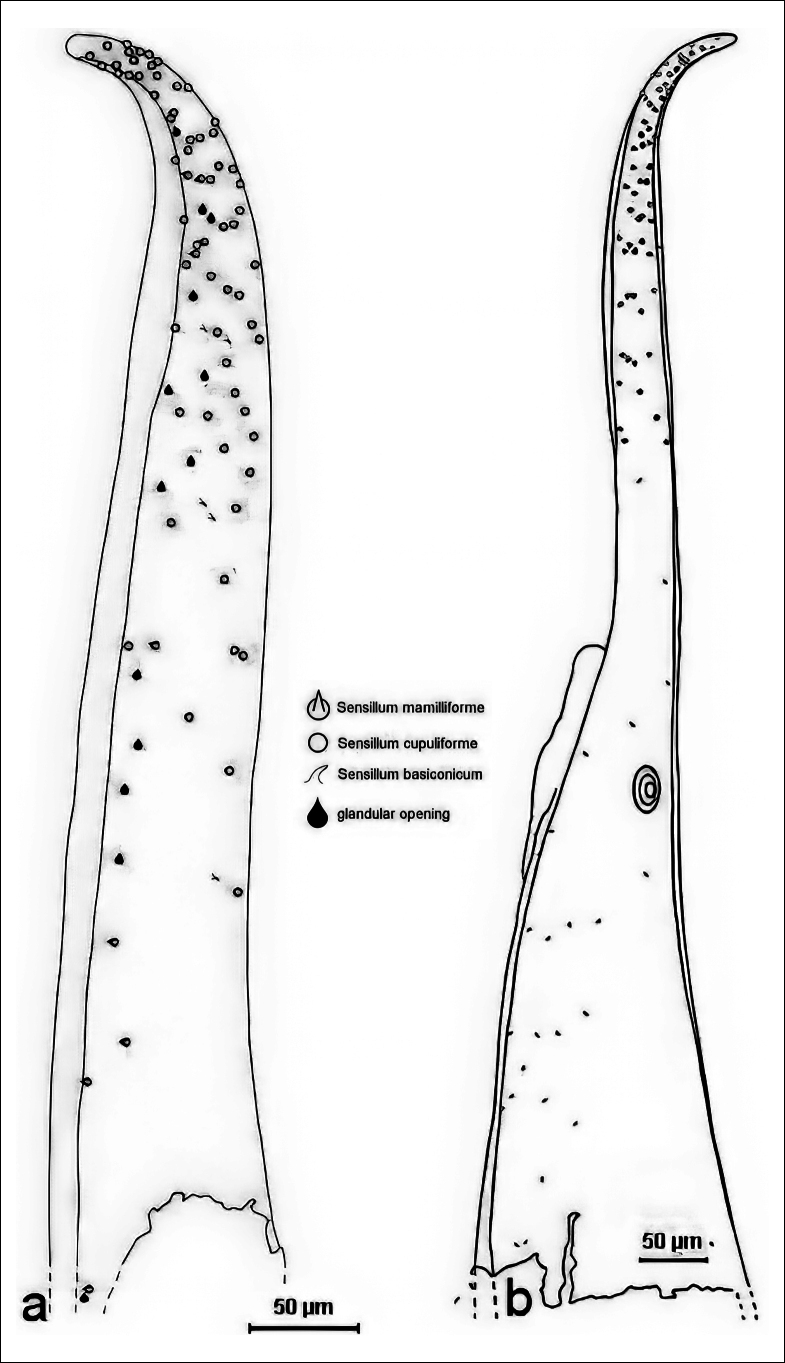
Distribution of sensilla on the lateral sides of the two median lobes. **a.** Left side; **b.** Right side. There is a membranous structure of unknown function, diameter c. 30 µm, only found in one specimen.

In addition to these putative sensilla, there are also numerous glandular openings, especially on the lower side of the median lobe. The distribution of these structures is shown in Figs 3–5, depicting the median lobes of four beetle males.

**Figure 6.**
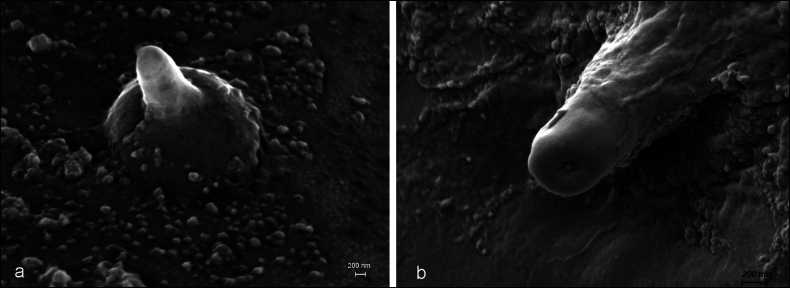
Sensilla mamilliformia, **a.** Under normal SEM; **b.** Under FE-SEM (showing the terminal pore).

On the upper side of the median lobe (Fig. 3), 11 sensilla mamilliforma could be counted, 35 s. basiconica, 54 s. cupuliformia and one glandular opening. On the lower side of another median lobe (Fig. 4) 31 s. mamilliformia, 9 s. basiconica, 52 s. cupuliformia and 15 glandular openings could be identified. The left side of a third median lobe (Fig. 5a) bears 8 s. mamilliformia, 5 s. basiconica, 59 s, cupuliformia and 13 glandular openings. On the right side of the fourth median lobe checked (Fig. 5b) 31 s. mamilliformia, 22 s. basiconica, 22 s. cupuliformia and 8 gland openings could be counted. The density of sensilla is highest near the apex, especially at the edge. The cupuliform sensilla were found in the highest numbers on the upper and lower sides, and even on the left side of one median lobe (Fig. 5a).

The tips of the s. mamilliformia appeared poreless under normal SEM (Fig. 6a). However, examination under high-resolution (field-emission-) SEM showed a depression most probably indicating a terminal pore in all sensilla inspected (Fig. 6b). Only a few gland openings were found on the upper side, and even in lateral view the majority was concentrated in the lower portion.

**Figure 7.**
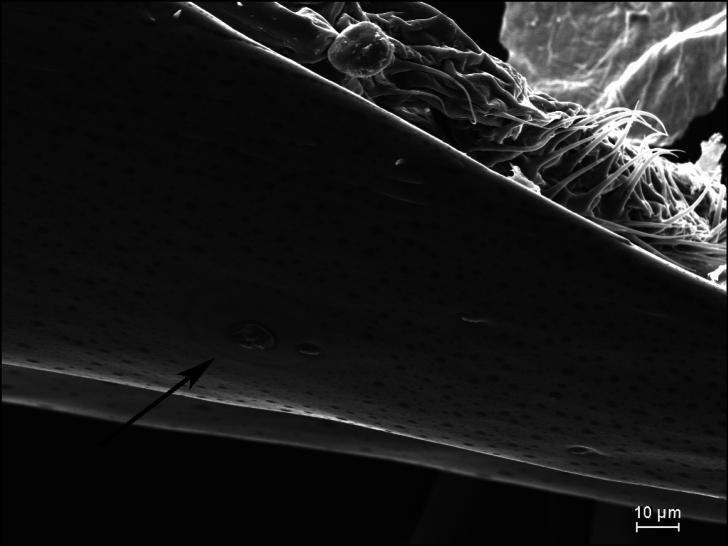
Detail of the right lateral side of one median lobe (drawn in whole in Fig. 5b). The arrow points to the enigmatic disk-shaped structure found only on this specimen.

One median lobe bears a disk-shaped structure on the lower right side (Figs 5b, 7), approximately below the ostium (the distal opening of the medial lobe through which the endophallus is everted during copulation). As it was only found in one of the nine specimens, it could have been caused by a developmental irregularity.

The structures here interpreted as sensilla basiconica are peg- or cone-shaped protrusions of the cuticle, always over a hollow or trough (Fig. 2c), sometimes standing upright, sometimes leaning over or into the hollow (see Fig. 7). Most sensilla basiconica are located near or on the outer face of the median lobe. Such structures are also found far more proximal than the other types.

## ﻿Discussion

*Agelastica alni* males have probably more than 200 sensilla of different types on the surface of their aedeagi. The number of sensilla counted in four specimens (Figs 3–5) indicates intraspecific variability and that there is no fixed spatial pattern.

Investigations by means of SEM can only reveal the external appearance of cuticular structures. Thus, labelling the structures here “sensilla” is only based on their similarity with already described sensilla. As the sensilla cupuliformia seem to have a pore, they are probably contact chemoreceptors. The bases of their domes are smoothly connected to the surrounding cuticle, without any trace of a membranous ring around the socket. Thus, they seem rigid and are therefore unlikely to perceive mechanical stimuli. Whether the so-called sensilla basiconica can be deflected is unclear, but the cuticular cones can certainly be deformed by mechanical forces. Thus, they can probably perceive mechanical pressure or vibrations, as for example, the digitiform sensilla of elaterid larvae (Zacharuk et al. 1977). If this should hold true, it would mean that male alder leaf beetles can perceive chemical and mechanical cues when the median lobe is intromitted in the female genital tract.

The structures here called “sensilla mamilliformia” appeared poreless at first sight. Since the dome-like elevated socket of the terminal peg or cone appears to sit in a ring-shaped depression indicated by the dark shadow around the base in Fig. 2a, they can probably be deflected mechanically and thus act as mechanosensors. However, by examination under high-resolution field-emission scanning electron microscopy, it turned out that they have a terminal pore, suggesting that they are contact chemoreceptors. They could be modified sensilla basiconica. But sensilla of this type “have dual chemo- and mechanosensitivity” (Zacharuk 1985: 38). Thus, it is well possible that male alder leaf beetles receive sensory input of both modalities through these sensilla. Nevertheless, as long as there are no transmission electron microscope (TEM) data available, let alone electrophysiological experiments, we can only infer per analogy that these structures are bimodal sensilla. Gnatzy and Tautz (2023: 207, fig. 9.11) mention a surprisingly similar sensillum on the wall of the pharynx of *Acherontia atropos* (Lepidoptera, Sphingidae) where they explicitly mention a terminal pore.

The single possible Sensillum campaniforme (Fig. 2d) bears a slight central depression that could be a covered pore. Since the aedeagus is formed during final metamorphosis and is never moulted, this mark cannot be an ecdysal pore (see Zacharuk 1985: 29). Possibly, this structure is no campaniform sensillum at all but a deformed sensillum cupuliforme.

The secretions escaping from the gland openings could act as a lubricant. Alternatively or additionally, they could contain signaling substances that the females could perceive with the help of contact chemosensors in the genital tract.

The results of the present study, together with the scattered findings published in the literature (Hammond 1972; Epila-Otara and Triplehorn 1990; Kim et al. 1999; Kim and Sota 2004, 2006; Hubweber and Schmitt 2006; Düngelhoef and Schmitt 2006, 2010; Kabaluk 2020), indicate that male beetles have sensilla on the median lobes of their aedeagus that make chemical and mechanical perception likely during copulation. The information perceived could serve different functions. Epila-Otara and Triplehorn (1990) hypothesise that male Platypodidae can avoid allospecific copulations by mechanically testing a potential mating partner. However, as Eberhard (1985) has convincingly explained theoretically and shown empirically in numerous cases, the morphology of copulatory organs is not determined by the lock-and-key principle in evolution, but is shaped by sexual selection. The case study of three species of *Macrodactylus* beetles (Coleoptera, Scarabaeidae) is a striking demonstration that neither lock-and-key, nor specific mate recognition, nor selection for species isolation could plausibly explain the morphological species-specificity of the male genitalia, but only the theory of female choice (Eberhard 1992). Since it is well confirmed that males of several beetle species studied so far court internally during copulation (Eberhard 1994, Eberhard and Kariko 1996), it seems reasonable to assume that the sensory equipment of the intromittent organ evolved in the context of copulatory courtship. The mechanoreceptors could, in the first place, function to aid the male in mechanically positioning his genitalia appropriately in order to carry out sperm transfer *per se* more effectively. They could also make it easier, or even possible, to place the intromittent part of the aedeagus at the appropriate sites inside the female body and thus optimise the effect of internal courtship. By perceiving the chemical characteristics in the female genital tract, the males presumably gain information on the mating status and receptivity of the female, and possibly also on the developmental state of the ova. Chaudhary et al. (2025) found that the mating status of both males and females of the ladybird *Menochilus sexmaculatus* (Fabricius, 1781) has a significant influence on the mating success of the last copulating male. The authors discuss if males may not only be able to differentiate between virgin and mated females, but also between singly-and multiply-mated females by modulating their ejaculate quality and quantity in relation to the female mating status. Assessing the mating status of a female will thus have direct consequences for the male’s fitness. It seems plausible that sensory input from inside the female genital tract can provide relevant information for this assessment.

Variation in the sites where particular sensilla occur, as suggested by the present results, is interesting in two contexts. In the first place, variation in the sites of sensilla will presumably result in perceptual “noise” or imprecision for the male. Lock-and-key ideas regarding genital evolution, especially those related to lock-and-key stimulation sensu Masley (2012), might predict relative invariance in the sites of sense organs, securing a more precise fit with the female structures. Secondly, analyses of receptor variation have been performed in female tactile sense organs of sepsid flies that perceive stimuli from species-specific male contact courtship structures by Asgari and Seiedy (2025), and it turned out that their observations support a female-choice- rather than the sexual-conflict-hypothesis. Consequently, a detailed analysis of the spatial distribution pattern of sensilla on the median lobes would also contribute to a deeper understanding of the evolution and functional roles of these structures in maximising reproductive success of both sexes.

From this follows that it would be advantageous for the male if sensilla were also on the parts of the male copulatory apparatus that are in even more intensive contact with the female body during copulation, especially on the endophallus. As this is a highly membranous structure, search for sensilla on its surface is considerably more difficult and requires TEM. There are only a few reports of sensilla on the endophallus wall of beetles based on SEM. Düngelhoef and Schmitt found one putative sensillum campaniforme on the surface of the endophallus of a *Lilioceris lilii* male (Düngelhoef and Schmitt 2006, fig. 14). Long and Tong (2025) present a photo of a sensory peg on the endophallus of a longicorn beetle.

It is also desirable to search for the presence of sensilla on and in the membranous wall of the female genital tract, especially the vagina and the bursa copulatrix. To my knowledge, there are hardly any such reports, one is given by Matsumura (2025, figs 2E, 4D). Nevertheless, it is highly probable that copulation in beetles is accompanied by genital communication between the mating partners and that aedeagal sensilla play a major part in this context.

## ﻿Additional information

### Author ORCIDs

Michael Schmitt https://orcid.org/0000-0001-7377-3643

### Data availability

All of the data that support the findings of this study are available in the main text.
